# An Estrogen Model: The Relationship between Body Mass Index, Menopausal Status, Estrogen Replacement Therapy, and Breast Cancer Risk

**DOI:** 10.1155/2012/792375

**Published:** 2012-01-16

**Authors:** Linda E. Green, Tuan A. Dinh, Robert A. Smith

**Affiliations:** ^1^Dominican University of California, 50 Acacia Avenue, San Rafael, CA 94901, USA; ^2^Archimedes, Inc., 201 Mission Street, San Francisco, CA 94105, USA; ^3^American Cancer Society, 250 Williams Street NW, Atlanta, GA 30303, USA

## Abstract

We present a mathematical model that lends support to the hypothesis that estrogen levels mediate the complex relationship between body mass index (BMI), menopausal status, estrogen-only hormone replacement therapy (HRT), and breast cancer risk. The model predicts a decrease in the relative risk of breast cancer of 3% per unit increase in BMI (kg/m^2^) for premenopausal women and an increase in the relative risk of 4% per unit increase in BMI for postmenopausal women who are not HRT users. When comparing postmenopausal women who use estrogen-only HRT to postmenopausal women who do not use HRT, the model predicts an increased risk of breast cancer associated with use of estrogen that diminishes with increasing BMI, with a relative risk of 1.6 for women with BMI of 18, 1.2 for women with BMI of 25, and 1.0 for women with BMI ≥ 30. Model predictions agree with data from five major epidemiological studies.

## 1. Introduction

The relationship between body mass index (BMI), menopausal status, and breast cancer risk is complex. In premenopausal women, higher BMI is associated with a decreased risk of breast cancer, while in postmenopausal women who do not use hormone replacement therapy (HRT) higher BMI is associated with an increased risk of breast cancer [1]. Use of HRT adds further complexity. Postmenopausal women who use HRT have an increased risk of breast cancer compared to postmenopausal women who do not use HRT [2, 3]. The increased risk of breast cancer associated with HRT use is attenuated in women with high BMI [3]. Furthermore, the increased risk of breast cancer associated with high BMI is not seen in women who use HRT [4]. 

Estrogen pathways provide a biological explanation for the relationship between BMI and breast cancer. For postmenopausal women, estrogen levels increase with increasing BMI, presumably because conversion of androgens to estrogen in adipose tissue is a primary source of estrogen [1, 5]. Estrogens have been shown to increase cell proliferation in normal and malignant breast tissue [2]. Therefore, it is regarded as biologically plausible that high levels of circulating estrogens increase risk of breast cancer, and this association has been observed in prospective studies [6, 7]. Since estrogen levels increase with BMI and breast cancer risk increases with estrogen level, higher estrogen levels have been hypothesized to be the mechanism behind the increase in breast cancer risk for postmenopausal women with high BMI [4, 8, 9]. The absence of an association between BMI and breast cancer risk in postmenopausal women who take HRT may be because elevated levels of circulating estrogens due to HRT use dwarf the elevated levels due to high BMI. Similarly, the attenuation of the increased risk of breast cancer associated with HRT use in women with high BMI may be because women with high BMI already have elevated levels of circulating estrogens [4, 8].

Estrogen pathways also are hypothesized to explain the relationship between BMI and breast cancer risk in premenopausal women [4, 10]. Studies of estrogen in premenopausal women are complicated by the fact that estrogen levels vary greatly during the menstrual cycle. However, an association between high BMI and low estrogen concentration is apparent when concentration is measured during the follicular phase of the menstrual cycle [10]. Lower levels of estrogen during the follicular phase correspond to a lower risk of breast cancer [11].

This qualitative explanation for the relationship between BMI, menopausal status, and breast cancer risk based on estrogen levels has been discussed in detail elsewhere [4, 8, 9, 12]. Other mechanisms, including insulin, insulin-like growth factors, leptin, and adiponectin, have also been proposed in the etiology of observed risk patterns [12, 13]. Among estrogens, free estradiol (estradiol not bound by albumin or sex hormone binding globulin) is the most biologically active form and has been associated with the greatest increase in breast cancer risk in premenopausal and postmenopausal women [6, 11].

In this study, we use a mathematical model to evaluate the hypothesis that free estradiol levels drive the association between BMI, menopausal status, estrogen-only hormone replacement therapy, and breast cancer risk. We focus our attention on estrogen-only HRT, also referred to as estrogen replacement therapy (ERT), rather than combined estrogen/progestin hormone replacement therapy. Combined hormone replacement therapy is associated with a greater increase in risk of breast cancer than ERT [14], suggesting the possibility of additional mechanisms at work. We assume the existence of a saturation level for postmenopausal women, above which additional amounts of free estradiol do not incur additional risk. We compare the predictions of the model to patterns of breast cancer risk observed in epidemiological studies and randomized controlled trials.

## 2. Materials and Methods

We performed a search of MEDLINE using the MeSH search terms: body mass index, estradiol, breast neoplasm, risk factors, premenopause, postmenopause, menopause, and hormone replacement therapy. We selected articles that reported quantitative relationships between BMI, HRT, and blood serum concentrations of free estradiol or between blood serum concentrations of free estradiol and breast cancer risk. We excluded articles that summarized data using fewer than three strata for BMI, articles on premenopausal women that did not report data on follicular phase estradiol levels, and articles on postmenopausal women based on fewer than 100 observations. We derived model inputs from published meta-analyses where available; in particular, reanalyses from the Endogenous Hormones and Breast Cancer Collaborative Group were the primary source for model inputs for postmenopausal women.

### 2.1. BMI and Postmenopausal Breast Cancer

We derived an exponential relationship between BMI and free estradiol concentration (*E*_2*F*_) from summary data from the reanalysis of eight prospective studies reported in Table 2 of Key et al. [9]. This reanalysis excluded women who were taking HRT at the time of blood collection. We estimated the median BMI for each reported BMI category from the BMI distribution for postmenopausal women in the National Health and Nutrition Examination Surveys (NHANES) [15]. We fit a least-squares line to data points representing median BMIs and mean log free estradiol concentrations (mol/L) for each BMI category, yielding the following equation:

(1) $$\begin{array}{c} E_{2F}=\exp\left(0.068847\cdot \text{BMI}-29.984\right). \end{array}$$

According to a reanalysis of nine prospective studies [6], the relative risk of breast cancer per doubling of free estradiol concentration among postmenopausal women who never used HRT was 1.50 (95% CI 1.22–1.85), giving the equation

(2) $$\begin{array}{c} RR={\left(\frac{E_{2F}^{1}}{E_{2F}^{2}}\right)}^{\log_{2}1.5}, \end{array}$$

where *RR* is relative risk and *E*_2*F*_^1^ and *E*_2*F*_^2^ are two different concentrations of free estradiol.

Combining (1) and (2) resulted in the following model for relative risk of breast cancer in postmenopausal women who are not HRT users as a function of BMI (relative to a reference BMI of 23):

(3) $$\begin{array}{c} RR={\left(\frac{\exp(0.068847\cdot \text{BMI}-29.984)}{4.6325\times 10^{-13}}\right)}^{\log_{2}1.5}, \end{array}$$

which simplifies to

(4) $$\begin{array}{c} RR=\exp\left(0.040273\cdot \text{BMI}-0.92628\right) \end{array}.$$

This represents an increase in relative risk by a factor of 1.04 for each unit increase in BMI (1 kg/m^2^).

Several studies have observed a plateau in breast cancer risk as a function of BMI for postmenopausal women with BMI ≥ 30 [9, 16, 17]. In order to capture this effect, we hypothesized the existence of a saturation level for postmenopausal women, above which additional amounts of free estradiol do not incur additional risk. A proposed saturation level of 7.50 × 10^−13^ mol/L of free estradiol corresponds to the concentration of free estradiol predicted for a woman of BMI = 30 according to (1). This assumption changed (2) for the relative risk of breast cancer associated with free estradiol to

(5) $$\begin{array}{c} RR={\left(\frac{\min(7.50\times 10^{-13},E_{2F}^{1})}{\min(7.50\times 10^{-13},E_{2F}^{2})}\right)}^{\log_{2}1.5} \end{array},$$

and (3) for the relative risk associated with BMI in postmenopausal women who are not HRT users to

(6) $$\begin{array}{cc} & RR\\ & ={\left(\frac{\min(7.50\times 10^{-13},\exp(0.068847\cdot \text{BMI}-29.984))}{4.6325\times 10^{-13}}\right)}^{\log_{2}1.5}. \end{array}$$

### 2.2. ERT and Postmenopausal Breast Cancer

We used the same framework to develop a model for the effect of ERT on breast cancer risk, under the hypothesis that this effect is mediated primarily through free estradiol level. The effect of ERT on estradiol level was modeled using summary data from the Nurses' Health Study, in which approximately 58% of women taking HRT used estrogen-only preparations [8]. Current users of HRT had higher free estradiol concentrations than nonusers. The ratio of free estradiol in current users compared to nonusers decreased with BMI and could be approximated by the equation

(7) $$\begin{array}{c} \text{ratio}=\exp\left(-0.023098\cdot \text{BMI}+1.2617\right) \end{array}.$$

We obtained an expression for the relative risk of breast cancer as a function of BMI in ERT users, relative to a BMI of 23, as follows. Let *E*_2*F*_^1^ and *E*_2*F*_^2^ represent the free estradiol levels corresponding to an unspecified BMI and a BMI of 23, respectively. From (1) and (7),

(8) $$\;\begin{array}{cc} E_{2F}^{1} & =\exp\left(-0.023098\cdot \text{BMI}+1.2617\right)\\ & \;\cdot \exp\left(0.068847\cdot \text{BMI}-29.984\right)\\ & =\exp\left(0.045749\cdot \text{BMI}-28.7223\right),\\ E_{2F}^{2} & =\exp\left(-0.023098\cdot 23+1.2617\right)\\ & \;\cdot \exp\left(0.068847\cdot 23-29.984\right)\\ & =9.6155\;\times \;10^{-13}. \end{array}$$

Substituting into (5) and simplifying yields the following equation:

(9) $$\begin{array}{cc} & RR\\ & ={\left(\frac{\min\left[7.50\times 10^{-13},\exp\left(0.045749\cdot \text{BMI}-28.7223\right)\right]}{7.50\times 10^{-13}}\right)}^{\log_{2}1.5}. \end{array}$$

Similarly, the equation

(10) $$\begin{array}{cc} & RR\\ & ={\left(\frac{\min[7.50\times 10^{-13},\exp(0.045749\cdot \text{BMI}-28.7223)]}{\min[7.50\times 10^{-13},\exp(0.068847\cdot \text{BMI}-29.984)]}\right)}^{\log_{2}1.5} \end{array}$$

models the relative risk of breast cancer associated with ERT use for women of a fixed BMI.

### 2.3. BMI and Premenopausal Breast Cancer

We constructed a similar model to describe the inverse relationship between BMI and breast cancer risk in premenopausal women. We derived an exponential relationship between BMI and follicular phase free estradiol concentration, using a least-squares fit to log-transformed data in Table 2 of Potischman et al. [10]

(11) $$\begin{array}{c} E_{2F}=\exp\left(-0.039851\cdot \text{BMI}-24.906\right) \end{array},$$

where *E*_2*F*_ represents follicular free estradiol.

We estimated the relative risk of breast cancer associated with a doubling of follicular free estradiol concentration as 1.7018 from Table 3 of Eliassen et al. [11], by fitting a line through the reference value to a log-log plot of relative risk by free follicular estradiol concentration. The slope of the line was 0.7671, so

(12) $$\begin{array}{c} 0.7671=\frac{\ln\left(RR_{1}\right)-\ln\left(RR_{2}\right)}{\ln\left(E_{2F}^{1}\right)-\ln\left(E_{2F}^{2}\right)} \end{array},$$

where *E*_2*F*_^1^ and *E*_2*F*_^2^ represent follicular free estradiol levels and *RR*_1_ and *RR*_2_ represent the corresponding relative risks of breast cancer, relative to the fixed reference value. Equation (12) simplifies to

(13) $$\begin{array}{c} RR={\left(\frac{E_{2F}^{1}}{E_{2F}^{2}}\right)}^{\log_{2}1.7018} \end{array},$$

where *RR* = *RR*_1_/*RR*_2_ represents relative risk of breast cancer for a follicular phase free estradiol concentration of *E*_2*F*_^1^ relative to *E*_2*F*_^2^.

Combining (11) and (13) and simplifying leads to the following model of premenopausal breast cancer relative risk as a function of BMI, relative to a BMI of 23.

(14) $$\begin{array}{c} RR=\exp\left(-0.030570\cdot \text{BMI}+0.70307\right) \end{array}.$$

This corresponds to a decrease in relative risk by a factor of 0.97 for each unit increase in BMI (1 kg/m^2^).

## 3. Results

The model of BMI and breast cancer reported here supposes that breast cancer risk associated with BMI is mediated primarily by free estradiol, with no other important causes. The model agrees with studies that measure relative risk of breast cancer directly as a function of BMI, lending support to this hypothesis. All of the data sources used in the comparisons that follow were independent of those used to build the model, with the exception of the Pooling Project [16], which overlapped slightly with the reanalyses [6, 9] used for model building; in addition, the hypothesized saturation effect was motivated in part by results from the Pooling Project (see Table 1).

### 3.1. Model Predictions and Validation for Premenopausal Women

**Figure 1:**
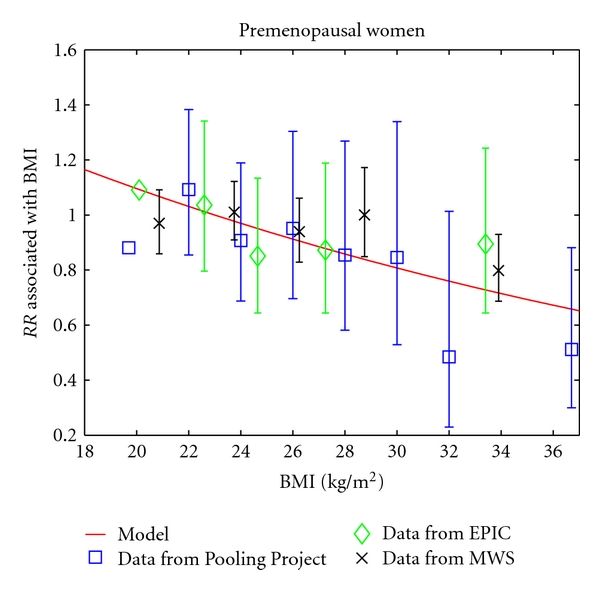
The model's prediction of the relative risk of breast cancer associated with BMI in premenopausal women is compared to the relative risks reported in three studies [16–18]. All relative risks are rescaled to use a BMI of 23 as the reference value. Error bars represent 95% confidence intervals and are missing from data points used as reference values in the original studies.

**Figure 2:**
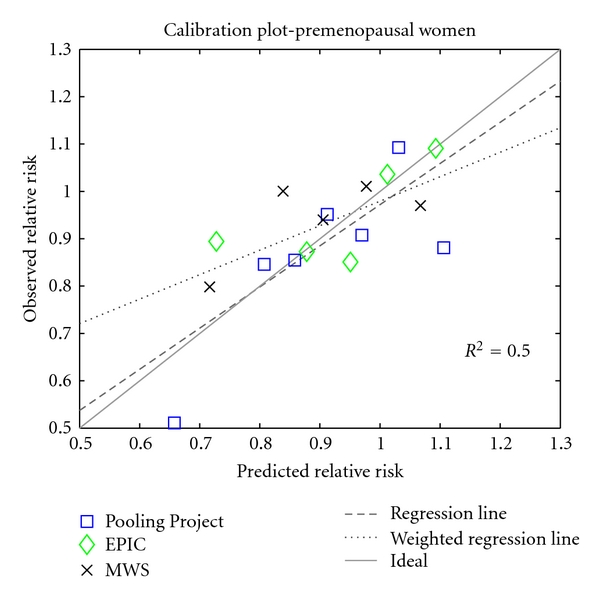
Calibration plot for the premenopausal breast cancer model, using observations from three studies [16–18]. The solid line indicates perfect calibration; the dashed line is a regression line (*y* = 0.8692 · *x* + 0.1028); the dotted line is a least-squares line, weighted by the number of observed breast cancer cases represented by each data point, an approximate measure of sample size (*y* = 0.5169 · *x* + 0.4623).

Figure 1 compares the model's predictions of relative risk of breast cancer associated with BMI in premenopausal women with relative risks reported in analyses from the Pooling Project [16], the European Prospective Investigation into Cancer and Nutrition (EPIC) study [17], and the Million Women Study [18]. In order to compare these studies, which used different BMI categories, we plotted midpoints of BMI categories. For the lowest and highest BMI category for each study, we estimated median BMIs from NHANES data [15]. We rescaled relative risks to correspond to a reference BMI of 23. This reference value was used because it represents a typical and healthy BMI towards the center of the BMI scale. Comparisons made with respect to a BMI of 23 are likely to reflect the overall trend of the data and are unlikely to be influenced by possible idiosyncratic effects of extreme BMIs. We used a calibration plot to quantify the agreement between model predictions and observed data (see Figure 2). The model captures the trend of the data well but predicts more extreme values of relative risk than the observed values.

For premenopausal women, the model finds a reduction in relative risk of 3% per unit increase in BMI (1 kg/m^2^). The Pooling Project reported an 11% reduction of relative risk per 4 kg/m^2^ increase in BMI, which is approximately a 3% reduction per unit increase in BMI [16]. An analysis of EPIC data reported a nonsignificant 2% reduction of relative risk per unit increase in BMI [17]. An analysis of the Million Women Study found a decrease in relative risk by a factor of 0.86 per 10 kg/m^2^ increase in BMI, which is about a 1.5% decrease per unit increase in BMI [18].

### 3.2. Model Predictions and Validation for Postmenopausal Non-HRT Users

**Figure 3:**
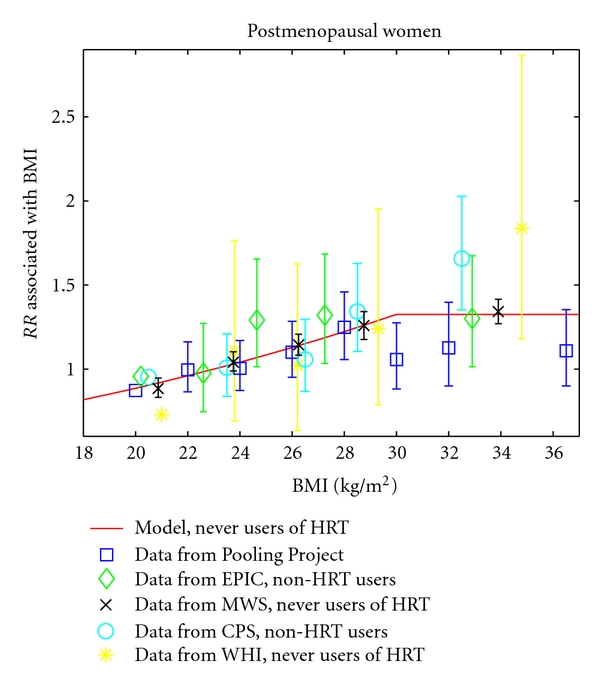
The model's prediction of the relative risk of breast cancer associated with BMI in postmenopausal women is compared to the relative risks reported in five studies [16–20]. All relative risks are rescaled to use a BMI of 23 as the reference value. Error bars represent 95% confidence intervals and are missing from data points used as reference values in the original studies.

**Figure 4:**
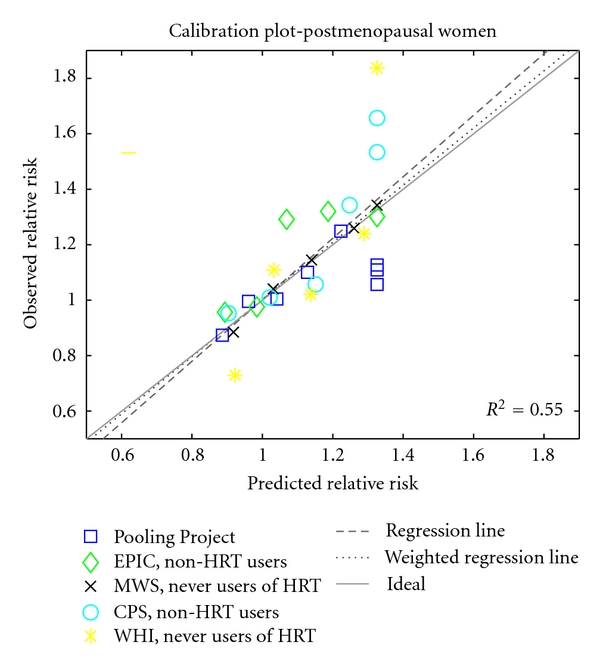
Calibration plot for the postmenopausal breast cancer model, using observations from five studies [16–20]. The solid line indicates perfect calibration; the dashed line is a regression line (*y* = 1.1088 · *x* − 0.1071); the dotted line is a least squares line, weighted by the number of observed breast cancer cases represented by each data point, an approximate measure of sample size (*y* = 1.0310 · *x* − 0.0285). The vertical clustering of points on the right side reflects the model's assumption that relative risk plateaus for BMI > 30.

Figure 3 compares the model's predictions of relative risk of breast cancer associated with BMI in postmenopausal women with relative risks reported in five analyses, based on data from the Pooling Project [16], the EPIC study [17], the Million Women Study [18], the Cancer Prevention Study-II Nutrition Cohort [19], and the Women's Health Initiative [20]. As for the premenopausal comparisons, relative risks for postmenopausal women were rescaled to correspond to a reference BMI of 23. Model predictions lie within the 95% confidence intervals for almost all of the studies and BMI categories. A calibration plot shows excellent agreement between model predictions and observed data (see Figure 4).

For postmenopausal women, the model finds a 4% increase in relative risk per unit increase in BMI for non-HRT users. An analysis of EPIC data reported a similar 3% increase in relative risk per unit increase in BMI for non-HRT users [17]. An analysis of the Million Women Study found an increase in relative risk by a factor of 1.4 per 10 kg/m^2^ increase in BMI for women who had never used HRT, which is about a 3% increase per unit increase in BMI [18]. The Pooling Project gave a smaller increase in relative risk of 7% per 4 kg/m^2^ increase in BMI, or slightly less than 2% per unit increase in BMI [16]. The smaller effect seen in the pooled analysis may be because the investigators did not stratify by HRT use.

### 3.3. Model Predictions and Validation for ERT

Although the hypothesis of a saturation level is speculative, it helps produce the correct interactions between BMI and HRT. With the saturation assumption, the model predicts no increase in relative risk of breast cancer associated with BMI for postmenopausal women who are taking ERT, since ERT use increases free estradiol levels beyond the hypothesized saturation level regardless of BMI, for BMI ≥ 18. This model prediction is consistent with observations from studies that show no increase in relative risk of breast cancer associated with BMI in HRT users [17, 19, 20].

**Figure 5:**
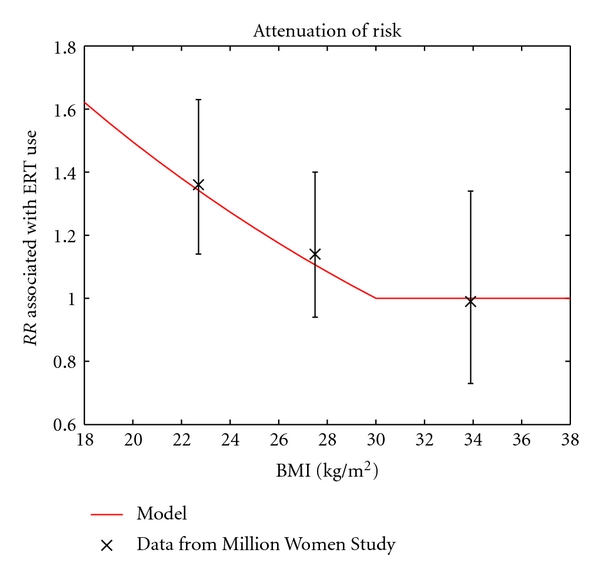
The model's prediction of the relative risk of breast cancer associated with ERT use agrees with data from the Million Women Study [14]. Error bars represent 95% confidence intervals.

When stratifying by BMI, the model predicts that the relative risk of breast cancer associated with ERT use should decrease from 1.6 in women with a BMI of 18 to 1.0 in women with a BMI of 30. This attenuation of the increased risk of breast cancer for HRT use agrees with data reported in Table  1 of Beral et al. [14], in which ERT users from the Million Women Study are compared to never users of HRT (see Figure 5). However, the model does not agree with results from the Women's Health Initiative, in which breast cancer risk for ERT users was nonsignificantly decreased in all BMI categories [21].

We used the model to predict an overall risk of breast cancer associated with ERT by taking the average relative risk over all values of BMI, weighted using the distribution of BMI among postmenopausal women in NHANES. The model predicts an overall relative risk of breast cancer associated with ERT use of 1.15. Other estimates of the relative risk of breast cancer associated with estrogen-only HRT include a nonsignificant decrease in relative risk of 0.82, (95% CI, 0.65–1.04) in the Women's Health Initiative [21], a relative risk of 1.3 (95% CI, 1.21–1.4) in the Million Women's Study [22], and a relative risk of 1.35 (95% CI, 1.21–1.49) in the reanalysis by the Collaborative Group on Hormonal Factors and Breast Cancer [3], in which 80% of HRT users used primarily estrogen-only preparations. The relative risk of 1.15 suggested by the model is plausible, given the range of estimates in these studies.

## 4. Discussion

The ability of this mathematical model to capture complex quantitative relationships suggests that estrogen levels are indeed responsible for the relationship between BMI, ERT, menopausal status, and breast cancer risk. Additional support for this hypothesis comes from an analysis of EPIC data and a reanalysis by the Endogenous Hormones and Breast Cancer Collaborative Group. Both analyses find that after adjusting for free estradiol concentration, the relative risk of breast cancer associated with BMI in postmenopausal women is not significantly different from 1.0 [9, 23].

The model proposes a threshold for free estradiol concentration in postmenopausal women, beyond which additional free estradiol does not incur additional risk of breast cancer. This hypothesis is speculative yet compelling, because it results in a model that neatly reproduces quantitative observations with respect to three phenomena: (i) the leveling off of breast cancer risk due to BMI for BMI > 30 in postmenopausal women who do not use HRT, (ii) the lack of effect of BMI on breast cancer risk for post-menopausal HRT users, and (iii) the attenuated effect of HRT on breast cancer risk for women with high BMI. Alternatively, it is possible that additional free estradiol beyond a certain concentration has a gradually decreasing effect on breast cancer risk rather than no effect. Available data on free estradiol and breast cancer risk could be used to test these hypotheses and to determine where the proposed threshold might lie.

The model has a number of limitations. First, the model was based on summary data from a number of different studies, which necessitated certain approximations. For example, summary data points, rather than individual level data, were used to estimate the relative risk of breast cancer per doubling of estradiol for premenopausal women. A second limitation is the variability in estradiol concentrations based on the assay used [6]. However, because the relative risk of breast cancer was represented as a function of doubling of free estradiol levels instead of absolute free estradiol levels, this variability should have minimal effect on the model's predictions of relative risk associated with BMI and ERT use. A third limitation is that the data from the Nurses Health Study used to model the effect of ERT on free estradiol levels combined different doses and forms of HRT [8]. Therefore, the model of the effect of ERT on free estradiol levels reflects average or typical use of HRT rather than any one particular dose or form. Some inaccuracy may result from using data that includes women taking combined HRT to represent the effect of estrogen-only HRT on estradiol levels.

Another limitation of the model for premenopausal women is that the underlying equations are based on limited data. Evidence from additional studies of hormone levels in premenopausal women was not used to build this model because the authors did not report follicular phase free estradiol concentrations [24–29]. However, several of these studies do show relationships between BMI and total estradiol concentrations in premenopausal women that are heterogeneous in nature. For example, Thomas et al. and Randolf et al. found a smaller decrease in follicular phase total estradiol concentration with increasing BMI than was found by Potischman et al. and used in the model [10, 24, 25]. Verkasalo et al. did not observe a trend of decreasing total estradiol concentration as a function of BMI, when using data from all phases of the menstrual cycle and adjusting for day of the cycle [26]. Emaus et al. found that multivariate adjusted total estradiol concentration in the saliva increased with BMI [27]. Because the concentration of free estradiol is primarily determined by the concentrations of total estradiol and sex hormone binding globulin, variations in the relationship between BMI and total estradiol levels found by different studies may indicate a more subtle relationship between BMI and follicular free estradiol level than is accounted for by the model. The possibility remains that other factors, such as obesity-related anovulation or alterations in hormones other than estrogen, may be important components of the explanation of the inverse association of BMI with premenopausal breast cancer risk.

The current model explains known relationships between BMI, ERT, and breast cancer risk in a mathematical framework that can be extended and refined. The model could potentially be used to estimate the effect of weight loss on breast cancer risk reduction, through estrogen pathway changes. In addition, it may be possible to incorporate physical activity into this estrogen model, since there is evidence that the protective effect of physical activity on breast cancer risk may be mediated through estrogen levels [30]. The model can easily be expanded to include total estradiol and sex hormone binding globulin, since concentrations of total estradiol and sex hormone binding globulin determine the concentration of free estradiol based on the law of mass action [31]. The model could also be improved by including combined HRT and the additional effect of progestin on breast cancer risk. Although combined HRT has a greater effect on breast cancer risk than estrogen-only HRT, the same patterns of interaction with BMI have been observed for both types of HRT [32]. Finally, the model could be refined to reflect the greater effect of estradiol on estrogen-receptor positive than estrogen-receptor negative breast cancer [33].

## 5. Conclusion

The estrogen-based mathematical model presented here predicts patterns in breast cancer risk that quantitatively agree with observations from epidemiological studies and randomized clinical trials, including the European Prospective Investigation into Cancer and Nutrition, the Million Women Study, the Cancer Prevention Study II, and the Women's Health Initiative. The model thus gives mathematical support to the hypothesis that estrogen levels are largely responsible for complex relationships between BMI, menopausal status, ERT use, and breast cancer risk.

## Figures and Tables

**Table 1:** Studies used to validate the model.

Study	Type of study	Baseline cohort size		Mean years of followup	Use for model validation
Pooling Project [16]	Pooled reanalysis of 7 cohort studies	Total*	337,819	3–8	Premenopausal and postmenopausal models

EPIC [17]	Prospective cohort study	Premenopausal	73,542	4.7	Premenopausal and postmenopausal models
		Postmenopausal			
		non-HRT users	79,030		

Million Women Study [14, 18]	Prospective cohort study	Premenopausal	63,153	5.4	Premenopausal, postmenopausal, and ERT models
		Postmenopausal			
		never users of HRT	392,757		
		current users of HRT	285,987		

CPS-II [19]	Prospective cohort study	Postmenopausal		8	Postmenopausal model
		non-HRT users	41,159		

WHI Observational Study [20]	Prospective cohort study	Postmenopausal		2.9	Postmenopausal model
		never users of HRT	32,547		

WHI Estrogen-Alone Trial [21]	Randomized controlled trial	Postmenopausal		7.1	ERT model
		non-HRT users	5,429		
		ERT users	5,310		

*Separate numbers of postmenopausal and premenopausal women included in the Pooling Project were unavailable.
